# Correlated missing linker defects increase thermal conductivity in metal–organic framework UiO-66†

**DOI:** 10.1039/d2sc06120a

**Published:** 2023-05-31

**Authors:** Meiirbek Islamov, Paul Boone, Hasan Babaei, Alan J. H. McGaughey, Christopher E. Wilmer

**Affiliations:** a Department of Chemical & Petroleum Engineering, University of Pittsburgh Pittsburgh Pennsylvania 15261 USA wilmer@pitt.edu; b Department of Chemistry, University of California Berkeley California 94720 USA; c Department of Mechanical Engineering, Carnegie Mellon University Pittsburgh Pennsylvania 15213 USA; d Department of Electrical & Computer Engineering, University of Pittsburgh Pittsburgh Pennsylvania 15261 USA

## Abstract

Thermal transport in metal–organic frameworks (MOFs) is an essential but frequently overlooked property. Among the small number of existing studies on thermal transport in MOFs, even fewer have considered explicitly the influence of defects. However, defects naturally exist in MOF crystals and are known to influence many of their material properties. In this work, we investigate the influence of both randomly and symmetrically distributed defects on the thermal conductivity of the MOF UiO-66. Two types of defects were examined: missing linker and missing cluster defects. For symmetrically distributed (*i.e.*, spatially correlated) defects, we considered three experimentally resolved defect nanodomains of UiO-66 with underlying topologies of bcu, reo, and scu. We observed that both randomly distributed missing linker and missing cluster defects typically decrease thermal conductivity, as expected. However, we found that the spatial arrangement of defects can significantly impact thermal conductivity. In particular, the spatially correlated missing linker defect nanodomain (bcu topology) displayed an intriguing anisotropy, with the thermal conductivity along a particular direction being higher than that of the defect-free UiO-66. We attribute this unusual defect-induced increase in thermal conductivity to the removal of the linkers perpendicular to the primary direction of heat transport. These perpendicular linkers act as phonon scattering sources such that removing them increases thermal conductivity in that direction. Moreover, we also observed an increase in phonon group velocity, which might also contribute to the unusual increase. Overall, we show that structural defects could be an additional lever to tune the thermal conductivity of MOFs.

## Abstract 2

Thermal transport in metal–organic frameworks (MOFs) is an essential but frequently overlooked property.

## Graphical abstract

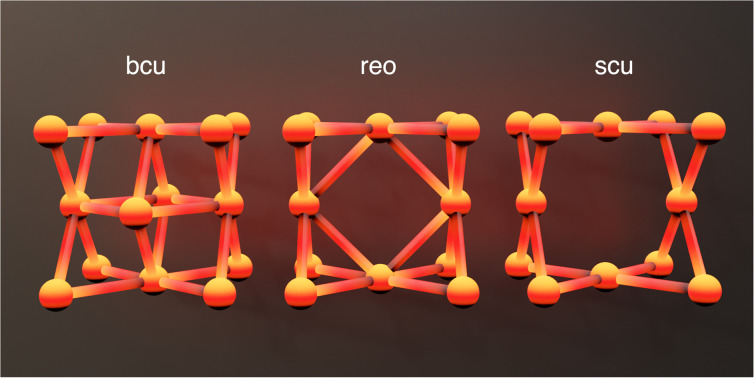

## Introduction

Metal–organic frameworks (MOFs) have attracted great interest due to their crystalline and nanoporous modular structure with ultrahigh internal surface areas that are tunable for a broad range of applications such as gas storage,^1–5^ chemical separations,^6–8^ species detection,^9–11^ catalysis,^12–14^ thermoelectrics,^15–19^ and drug delivery.^20–22^ MOFs are constructed from metal clusters and organic ligands, allowing scientists to precisely design pore chemistry and geometry *via* a reticular chemistry approach.^23–25^ Thermal transport in MOFs is a critical property that impacts the figure of merit (ZT) in thermoelectric applications that require low thermal conductivity.^15–19^ In gas adsorption applications (where high thermal conductivity is desired), thermal transport affects the adsorbate system's capacity, which depends on its ability to disperse the exothermic heat produced when gas is rapidly adsorbed. Despite its significance in practical implementations, the thermal transport properties of MOFs have not been extensively studied.^26–42^ Past work focused on the influence of framework architecture,^36^ pore size and shape,^37^ interpenetration,^38^ node-linker chemistry,^39^ guest–host interactions,^40^ and physisorbed^41,42^ and chemisorbed^43^ adsorbates on the thermal conductivity of MOFs. However, the presence of defects was often neglected in many studies on MOF thermal transport, although various types of defects are intrinsically present, even in neatly synthesized MOF crystals at finite temperatures.^44^

Defects in MOFs have captivated researchers due to their ability to enhance the various performance of MOFs, such as catalytic activity, improved adsorption, electrical conductivity, *etc.*^45^ Thus, researchers found ways to artificially introduce defects with controlled densities *via* so-called defect engineering into a parent framework to modify the functionalities of MOFs.^46^ The two widely applied synthetic routes for purposefully creating defective MOFs are the “*de novo*” synthesis and the postsynthetic treatment. In the former, a large amount of modulators (*i.e.*, monocarboxylic acid) is added along with linker molecules to facilitate the speed of crystallization and, thus, the formation of defects.^47,48^ In the latter, the defect sites are incorporated into the parent structure after synthesizing MOFs using modified linkers or acids.^49,50^

**Fig. 1:**
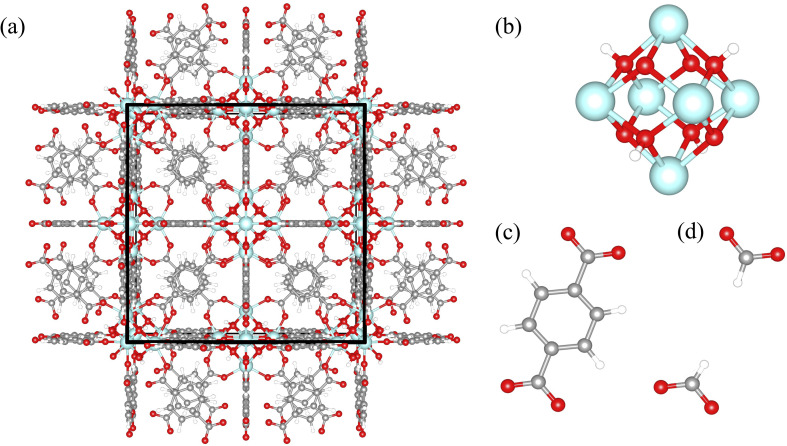
(a) Structure of UiO-66; a unit cell is marked with the black frame; (b) hexanuclear clusters, containing the six Zr atoms, the four μ_3_-O atoms, and the four μ_3_-OH groups; terephthalate (BDC) linker before (c) and after (d) linker removal. The color code for the atoms is as follows: zirconium (cyan), carbon (grey), oxygen (red), and hydrogen (white).

The MOF UiO-66 (Universitetet i Oslo) is recognized as a prototypical model system of defective MOFs (Fig. 1a).^53^ Since its discovery by Lillerud's group in 2008, it has been a focus of extensive MOF research due to superior mechanical and thermal stability, easy synthesis, tunability, and functionality, primarily due to controlled defects.^54^ UiO-66 [Zr_6_(μ_3_-OH)_4_(μ_3_-O)_4_(C_8_H_4_O_4_)_6_] is constituted by hexanuclear clusters (Fig. 1b) containing the six Zr atoms (or Hf,^55^ U,^56^ and Ce^57^ atoms) in octahedral geometry, the four μ_3_-O atoms, and the four μ_3_-OH groups, bridged with twelve terephthalate (BDC) ligands (Fig. 1c) (fcu topology, 12-coordinated nodes).^53^ An interesting feature of UiO-66 is that the inorganic component, Zr_6_(μ_3_-OH)_4_(μ_3_-O)_4_, can be dehydrated reversibly by removing the μ_3_-OH groups *via* heating to high temperatures.^58^ The hydroxylated and dehydroxylated states can occur reversibly as water molecules are released or taken up, respectively. The most widely studied form is the hydroxylated form of UiO-66, which is known to be more stable at room temperature and in the presence of water, which was used in this work.^54^

**Fig. 2:**
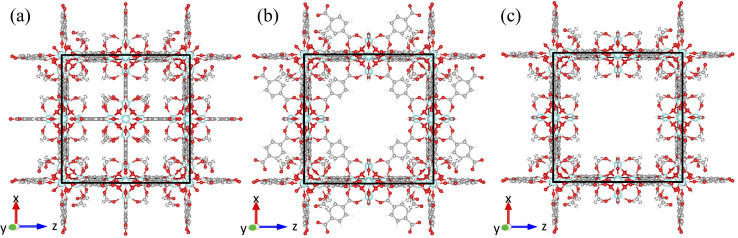
Illustrations of three correlated defect nanoregions; unit cells are marked with the black frame; (a) missing linker defects (bcu topology): all clusters are 8-connected; (b) missing cluster defects (reo topology): all clusters are 8-connected; (c) missing cluster defects (scu topology): corner clusters are 8-connected and edge clusters are 4-connected. The color code for the atoms is as follows: zirconium (cyan), carbon (grey), oxygen (red), and hydrogen (white).

There are two known types of point defects in UiO-66: missing linker^51^ and missing cluster defects.^59^ The first observation of the missing linker defects in UiO-66 was found to occur as an absence of a single linker out of twelve linkers connected to a cluster.^53^ Cliffe *et al.* reported the first observation of missing cluster defects in UiO-66 and found that missing cluster defects, where the Hf_6_ node with all twelve coordinated terephthalate linkers was missing from the framework, form correlated defect nanoregions.^60^ The correlated missing cluster defect nanoregions have a reo topology, where nodes are 8-connected instead of 12-connected (Fig. 2b). Perhaps more importantly, they demonstrated that structures with correlated disorder exist in the UiO-66 family and that those defects can be synthetically controlled. Subsequently, the presence of correlated missing cluster nanodomains was confirmed through the use of low-dose transmission electron microscopy and electron crystallography by Liu *et al.*,^61^ as well as scanning electron diffraction by Johnstone *et al.*^62^ Furthermore, Liu *et al.* also reported a different correlated missing cluster nanodomain, where the face-on terephthalate linkers that are not coordinated to the missing cluster but surrounding were also missing. It has an underlying net of scu topology, where some nodes are 8-connected and some are 4-connected (Fig. 2c). Other than correlated missing cluster nanoregions, they showed that missing linker defects could also form correlated nanoregions. It has the body-centered tetragonal structure (bcu net), where all face-on terephthalate linkers are missing from the parent framework when facing to the <001> zone axis (Fig. 2a).^61^

Several studies have investigated the introduction of exclusive missing linker and missing cluster defects in UiO-66. In the synthesis of UiO-66, Bueken *et al.* utilized a labile linker known as *trans*-1,4-cyclohexane-dicarboxylate.^51^ Through thermolysis, they were able to introduce exclusive missing linker defects in the resulting UiO-66. In a recent study, Feng *et al.*^52^ proposed a novel synthesis technique for the exclusive creation of missing cluster defects in UiO-66. Specifically, they utilized a mixed-metal (Zn, Zr)-UiO-66 and performed a soft acid wash that selectively removed the Zn nodes, resulting in Zr-UiO-66 with missing cluster defects.

Both missing linker and cluster defects are known to alter various properties of UiO-66. For instance, increasing the density of defects was found to decrease the thermal stability and water resistance but enhance the adsorption and catalytic activities.^63,64^ However, it is still unknown how missing linker and cluster defects affect the thermal conductivity of well-studied UiO-66. Since controlling the density of missing linker and missing cluster defects are valuable means of tuning material properties, understanding how those defects alter the thermal conductivity is necessary, especially for gas adsorption and catalysis applications, where rapid removal of exothermic thermal energy can be critical. In this work, we focused on the influence of randomly arranged missing linker and cluster defects on the thermal conductivity of UiO-66. Furthermore, we also reported the thermal conductivity of three correlated defect nanoregions (bcu, reo, and scu nets). We observed that both missing linker and cluster defects reduce the thermal conductivity at a different rate if randomly spread across the framework. Unexpectedly, we found that correlated missing linker defects (bcu net) showed improved thermal conductivity compared to a pristine UiO-66. Particularly, the thermal conductivity exhibited anisotropy, where it increased in the direction perpendicular to the removed face-on linkers but decreased in the other two directions.

## Methodology

We calculated the thermal conductivity of various defective UiO-66 structures with different defect densities using molecular dynamics (MD) simulation and the Green–Kubo method. The Green–Kubo formula is given by^65^1
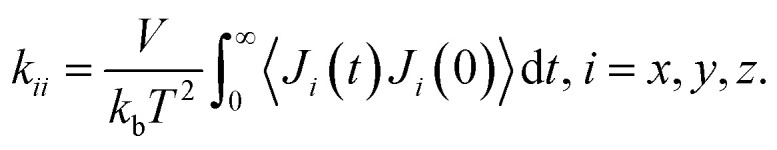
In eqn (1), 〈*J*_*i*_(*t*)*J*_*i*_(0)〉 is the heat flux autocorrelation function (HCACF), which represents how strong the correlation is between heat fluxes at time zero and *t*. *k*_b_ is the Boltzmann constant, *V* is the simulation cell volume, and *T* is temperature. To estimate the *i*-th diagonal component of the thermal conductivity tensor, the HCACF was integrated over a selected correlation time interval.

The open-source molecular dynamics code LAMMPS^66^ was used for all thermal conductivity simulations. We used a version of LAMMPS that can correctly calculate the heat flux for many body terms.^67^ The computational model for pristine UiO-66 was a cubic 3 × 3 × 3 supercell with a length of 62.1 Å in each dimension.^54^ There were 12 312 atoms, 648 terephthalate organic linkers, and 108 metal clusters in the 3 × 3 × 3 UiO-66 system. We used the recently developed MOFUN software to generate all defective UiO-66 structures and to create all three correlated defect nanoregions.^68^ MOFUN is an open-source Python package for searching for instances of a molecular substructure (*e.g.*, BDC linker) and replacing some of them at a given fraction with another pattern (*e.g.*, defective BDC linker capped with formate). For instance, to generate defective UiO-66 structures with randomly distributed missing linker defect sites, we first searched for all instances of the BDC linker in the UiO-66 structure. The search pattern (BDC linker) and its instance in the structure should share the same set of atom elements and the same relative atom positions (within a tolerance) for an instance to be a match. Then, a certain fraction of randomly selected linkers is replaced with the replacement pattern (defective BDC linker) by first rotating the replacement pattern to align with the match. Finally, selected matched atoms of the search pattern are deleted from the structure after inserting the replacement atoms. The same procedure is applied to introduce randomly arranged missing cluster defects as well as correlated defects.

The extension of the UFF force field^69^ that was specifically developed for MOFs (UFF4MOF)^70^ was used to simulate bonded interactions. We implicitly assumed that only phonons and no electrons contributed to thermal conductivity in UiO-66. In the MD simulation, we used a timestep of 0.5 fs for all structures. Initially, to set the temperature to 300 K, the canonical constant-volume-constant-temperature (NVT) ensemble was run for 500 ps. After that, we equilibrated structures using the microcanonical constant-volume-constant-energy (NVE) ensemble for 100 ps. Finally, we calculated the HCACF every five timesteps during the final NVE production that ran for 500 ps. We used a correlation length of 2 × 10^5^ in the HCACF. In all directions, we applied periodic boundary conditions. We used 16 independent simulations for each structure with different random initial velocity distributions to obtain a sufficient statistical sampling of phase space.

Because the unit cell of the UiO-66 is cubic, we refer to the *a*, *b*, and *c* crystallographic vectors as *x*, *y*, and *z* for convenience. In each simulation, we calculated the diagonal terms of the thermal conductivity tensor, each representing the *x*-, *y*-, and *z*-directional thermal conductivity. Since structures with random defects did not exhibit anisotropy, we averaged all three directional thermal conductivity values to obtain a single isotropic value. For all correlated defects we considered each directional thermal conductivity value separately because they displayed anisotropic thermal conductivity. To study the influence of density of randomly induced missing linker and missing cluster defects on the thermal conductivity, we considered defect densities between 0–50%, in 1% increments between 0–5%, and in 5% increments between 5–50%. For each defect concentration of random defects, three independent defective structures with the random missing linker or missing cluster defects were generated. The average thermal conductivity was obtained by averaging their respective thermal conductivities. For missing cluster defects, in addition to the Zr_6_ cluster, all 12 linkers connected to the cluster were removed.^60–62^ The missing cluster defect density was based on the number of removed clusters, not linkers. Upon generation, some organic linkers were left without bonded connections with the framework at higher missing cluster defect concentrations. Hence, we also removed them to make the structures more realistic. After the linker removal, we terminated the bonds with formate for all random and correlated defects, as suggested by experiments.^61^ However, we acknowledge that the nature of terminating bonds may be highly dependent on the specific synthesis conditions and materials being used. The terephthalate ligand with and without a defect is illustrated in Fig. 1c and d.

## Results and discussion

### Thermal conductivity of randomly arranged defects

**Fig. 3:**
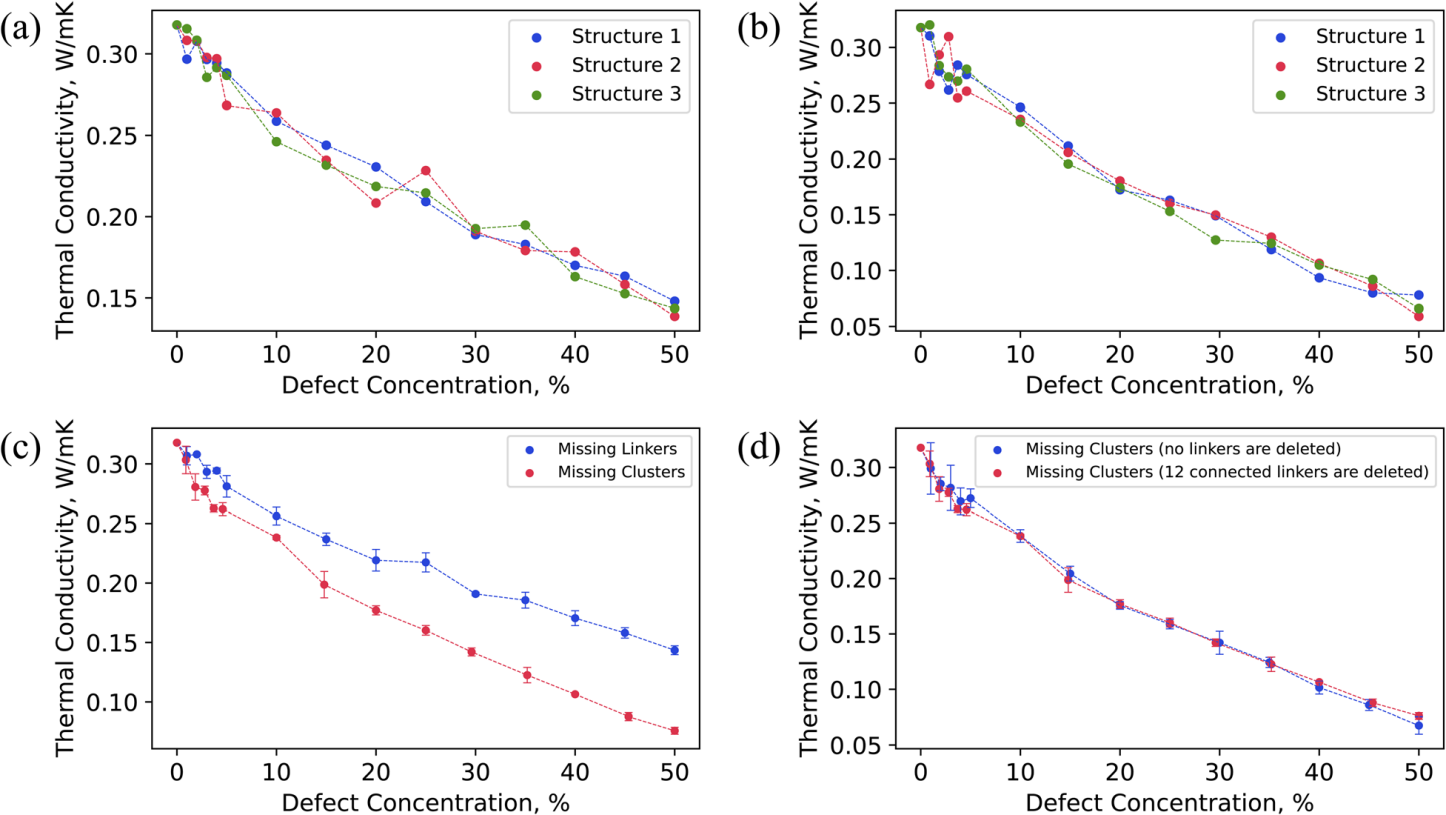
Thermal conductivity of UiO-66 (3 × 3 × 3 supercell) as a function of (a) randomly arranged missing linker defect density; (b) randomly arranged missing cluster defect density; (c) both randomly arranged missing linker and missing cluster defect densities. The lines illustrate the average thermal conductivity of three defective structures with the same defect density (averages of the data in (a) and (b), respectively); and (d) randomly arranged missing cluster defect densities (with and without deletion of all 12 linkers connected). In (a) and (b), each structure corresponds to a different spatial distribution of defects.

Fig. 3 illustrates that thermal conductivity (*k*) decreased as the concentration of missing linker and missing cluster defects increased, as expected. According to the classical phonon-gas model, this could possibly be due to increased phonon-defect scattering and change in volumetric heat capacity caused by mass density change.^71^ The calculated *k* of pristine UiO-66 was 0.32 W m^−1^ K^−1^. The pristine UiO-66 displayed isotropic behavior, where the *k* was the same in all crystallographic directions. This is due to the isotropic nature of the UiO-66 structure, where the heat transfer pathways are equivalent in all three crystallographic axes. In the past, the measured and molecular-dynamics calculated *k* of UiO-66 was 0.11 W m^−1^ K^−1^ and 0.87 W m^−1^ K^−1^, respectively.^32,33^ This measurement, however, was on compacted powder samples at varying water contents using a transient-hot-wire method, and the thermal conductivity of bulk UiO-66 crystals was derived using a biporous effective medium approximation (BEMA). It is worth mentioning that MOF powders are expected to have lower thermal conductivity due to void spaces and interparticle resistance relative to the bulk crystalline value. The corrected thermal thermal conductivity of bulk crystals using BEMA was still lower than our calculated value, which could be due to presence of defects at large amounts, as the characterization consisted of a single PXRD overlaid on an SEM of cubic crystals of UiO-66.^32^ In the previous molecular dynamics simulations, the discrepancy could be due to a different custom-developed interatomic potential and a version of LAMMPS that did not have the correct heat flux implementation for many-body potentials.^67^ The *k* dependency on defect concentration was consistent across all three randomly generated defect samples, each corresponding to a different spatial distribution of defects but the same defect concentration. However, all three structures with the same defect density had slightly different thermal conductivity values (within the range of uncertainty), indicating that not only the defect density but their spatial arrangement impacts the thermal conductivity in UiO-66.

As shown in Fig. 3c, the missing cluster defects were more effective in reducing the *k* than the missing linker defects. Especially at higher defect densities, the difference in the *k* values between the two types of defects was substantial. This could be because when each cluster was removed, all twelve coordinated linkers were removed from the structure, resulting in a more sparse framework. Each missing cluster defect density corresponds to some higher missing linker defect concentration. For instance, 25% missing cluster defect density corresponds to ∼45% missing linker defect density when considering only the number of removed linkers, as shown in Fig. S5b.† The corresponding thermal conductivities were 0.14 W m^−1^ K^−1^ and 0.16 W m^−1^ K^−1^ for missing linker and cluster defects, respectively. Although 25% missing cluster defect density is equivalent to removing 27 clusters in addition to removing linkers from the 3 × 3 × 3 UiO-66 supercell, the *k* values were similar. Although the number of deleted linkers was the same in both cases, only linkers that were connected to deleted metal clusters were removed for missing cluster defects. In contrast, defect sites of removed linkers were distributed throughout the structure for missing linker defects, leaving all clusters in the structure. Therefore, removing those disconnected clusters that resulted from the absence of surrounding twelve linkers did not further reduce the thermal conductivity since they no longer acted as heat transfer agents.

To further investigate the impact of missing cluster defects on thermal conductivity, we analyzed the thermal conductivity of defective UiO-66 structures under varying concentrations of missing linker and missing cluster defects, with the missing linker defect concentration serving as the *x*-axis (Fig. S5a†). The resulting curves exhibited similar trends, indicating that the primary cause of decreased thermal conductivity in the case of missing cluster defects is the removal of surrounding 12 linkers. Additionally, we conducted thermal conductivity calculations for defective structures featuring missing cluster defects, in which we removed metal nodes without removing the surrounding 12 linkers. Interestingly, the two curves displayed a similar decrease in thermal conductivity with respect to defect concentration, as shown in Fig. 3d. We hypothesize that this is because eliminating the metal nodes disrupts heat transfer pathways that depend on them, thus leaving the 12 linkers inactive in terms of heat conduction.

In the past, we investigated the influence of missing linker defect concentration on the *k* in another well-known MOF, HKUST-1.^71^ Missing linker defects significantly reduced the *k* in HKUST-1, even at lower concentrations. For example, 5% defect density caused a 70% drop in *k*. In UiO-66, the *k* dropped by only 12% at 5% missing linker defect concentration, whereas 50% defect density caused a 55% decrease in *k* compared to the defect-free UiO-66. In other words, 5% missing linker defect density resulted in a greater reduction of *k* in HKUST-1 than 50% defect density did in UiO-66. A significantly larger drop of *k* in HKUST-1 could be due to higher starting *k* (1.65 W m^−1^ K^−1^) for the pristine case in HKUST-1 compared to the pristine *k* in UiO-66 (0.32 W m^−1^ K^−1^). Alternatively, this might be due to UiO-66 having 12-coordinated nodes with six heat transfer pathways that go through them, allowing the remaining linkers after incorporating defects to serve as conduits. This might not be the case in HKUST-1, where nodes are 4-connected with only two heat transfer pathways. Removing even a small fraction of linkers in HKUST-1 deactivated many heat transfer pathways, significantly reducing the *k*. Moreover, due to UiO-66 possessing the highest possible coordination (twelve coordination), some linkers might conduct heat in a particular direction but act as scattering sources in other directions. Consequently, removing those linkers could benefit thermal conductivity in some cases. This could also explain why missing linker defects did not substantially reduce the *k* in UiO-66. Interestingly, the spatial arrangement of randomly occurring linker defects did not affect *k* in HKUST-1, which is in contrast to UiO-66. This difference can be attributed to the fact that UiO-66 has a 12-coordination, which provides more options for defect distribution within the framework at the same defect density compared to the 4-coordination in HKUST-1. In MOFs with low coordination, the density of defects can be the determining factor for *k*, rather than the spatial configuration of defects. However, in MOFs with high coordination where spatial arrangement also plays a role, it is reasonable to expect that different spatial configurations of defects at the same concentration may exhibit slightly different values of *k*.

### The thermal conductivity of correlated defect nanoregions

**Fig. 4:**
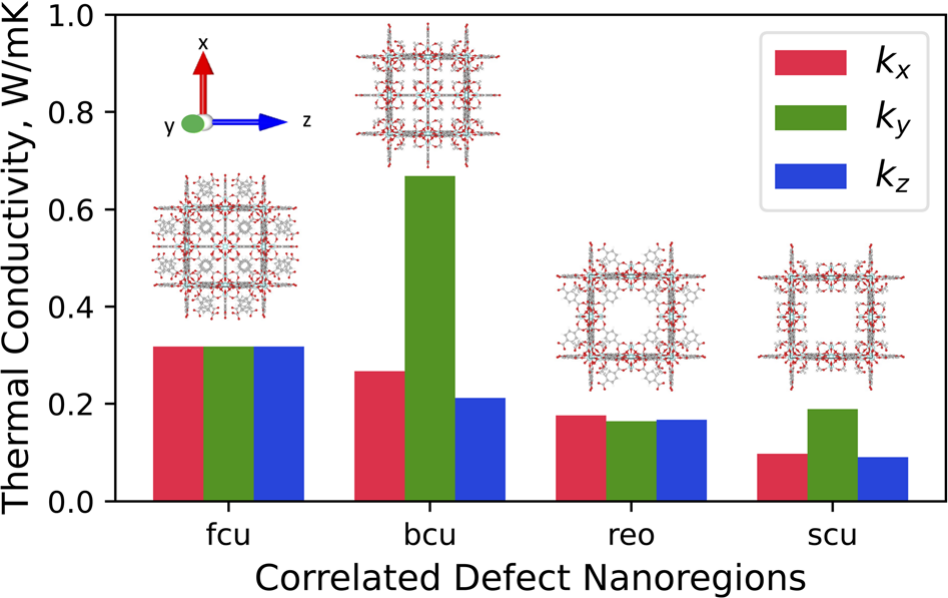
Directional thermal conductivity values of pristine UiO-66 (fcu) and various correlated defect nanoregions of UiO-66 (bcu, reo, and scu).

The *x*-, *y-*, and *z*-directional *k* values of pristine UiO-66 and three correlated defect nanoregions are illustrated in Fig. 4. In correlated missing linker defects (bcu topology), where eight face-on linkers were missing from the unit cell when facing the *y*-axis, the *k* decreased in the *x*- and *z*-directions but increased in the *y*-direction, exhibiting anisotropy (*k*_max_/*k*_min_ = 3.2). The *x*-, *y*-, and *z*-directional *k* values were 0.27, 0.67, and 0.21 W m^−1^ K^−1^, respectively. The *k* in the *y*-direction was more than twice of *k* of pristine UiO-66 (0.32 W m^−1^ K^−1^). This corresponds to 33% missing linker defect density (8 out of 24 linkers were missing from the unit cell). To compare, the 35% randomly arranged missing linker defect density had an average *k* of 0.19 W m^−1^ K^−1^, which was about two times smaller than correlated defects, demonstrating the importance of spatial configuration of defects at the equivalent defect density. This suggest that defects can be tuned not just to suppress the heat conduction but also to improve it. These correlated defects caused the mass density to drop from 1.25 g cm^−3^ in pristine to 1.14 g cm^−3^ in defective UiO-66 (bcu). The increase of *k* in the *y*-direction can be ascribed to removing all linkers perpendicular to it, which acted as the phonon-scattering source in the *y*-direction in the pristine UiO-66.^72^ Removing them reduced phonon scattering and hence increased *k*. However, those removed linkers served as heat transfer pathways in the *x*- and *z*-directions since they were posing parallel to them, and excluding them caused a reduction of *k* in the respective directions.

The thermal conductivity showed isotropic behavior in correlated missing cluster defect nanoregion (reo topology), where a single cluster with all 12 connected linkers was missing from the unit cell. It had *k* of 0.17 W m^−1^ K^−1^, almost two times lower than that of pristine UiO-66. That is because half of the linkers, and thus half of the heat transfer conduits, were missing in each dimension compared to pristine UiO-66. The structure had a mass density of 0.85 g cm^−3^, and 25% of clusters were absent. The random missing cluster defects showed similar thermal conductivity (0.16 W m^−1^ K^−1^) at the 25% defect density. Finally, in another correlated missing cluster defect nanoregion (scu topology), where face-on four linkers were missing from the unit cell of the previous structure (reo topology) when facing the *y*-axis, the *k* again exhibited anisotropy (*k*_max_/*k*_min_ = 2.1). The *x*-, *y*-, and *z*-directional *k* values were 0.10, 0.19, and 0.09 W m^−1^ K^−1^, respectively. Similar to bcu topology, the *k* slightly increased in the *y*-direction perpendicular to removed linkers and decreased in the *x*- and *z*-directions with respect to the reo topology. Again, eliminating those linkers reduced the phonon scattering in the *y*-direction and invalidated half of the heat transfer pathways in the *x*- and *z*-directions. Thus, in the *x*- and *z*-directions, the *k* was about two times lower than that of the reo structure. This defective structure had the lowest density among all correlated defect nanodomains (0.8 g cm^−3^). Unlike in the bcu topology, the average *k* did not improve compared to the reo topology. We believe this is because the structure with correlated defects (reo) was already sparse, where the nodes were 8-connected. Removing additional linkers did not improve the *k* substantially in the *y*-direction but reduced it significantly in the other two dimensions. Thus, we note that the number of parallel and perpendicular linkers to each dimension plays a crucial role in dictating the directional thermal conductivity in UiO-66. The resulting *k* imposed by correlated defects depends on the number of remaining linkers and the relative decrease and increase of the directional *k* values. We point out that real UiO-66 crystals might simultaneously contain all three correlated and randomly distributed defects. Thus, their thermal conductivity depends on their relative amount and micro-level spatial distribution.

**Fig. 5:**
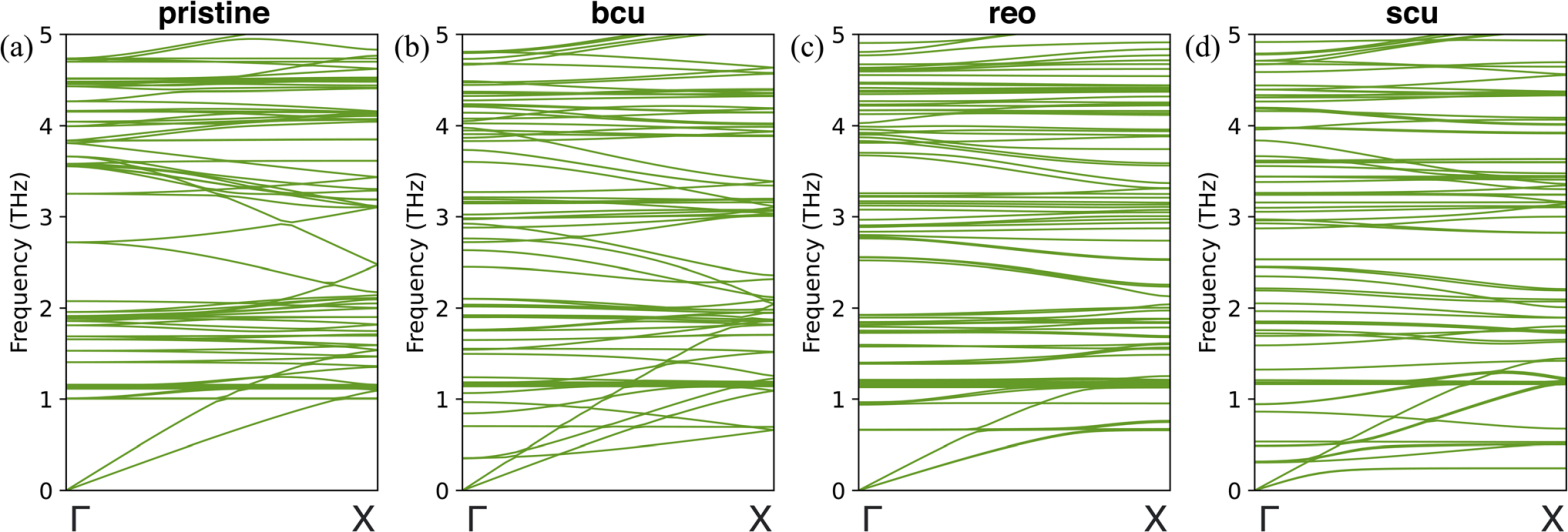
Phonon dispersion curves in the [010] direction (*y*-direction), covering the 0 to 5 THz frequency range, for (a) pristine UiO-66; (b) UiO-66-bcu; (c) UiO-66-reo; and (d) UiO-66-scu. The maximum frequency observed was 116 THz.

To gain further insight into the changes in the thermal conductivity of UiO-66 due to correlated defects, we performed harmonic lattice dynamics calculations using GULP.^73^ These calculations allowed us to investigate the effects of three correlated defects (bcu, reo, and scu) on the phonon dispersion curves, which are directly related to heat transport properties. We analyzed the phonon band structures of pristine UiO-66 and correlated nanodomains of UiO-66 along the [100], [010], and [001] directions, as shown in Fig. 5 and S9–S12.† Our results show that the phonon dispersion curves of pristine UiO-66 are isotropic, with three acoustic phonon branches (longitudinal and two degenerate transverse) and no optical branches below 1 THz (Fig. 5a). In contrast, the phonon dispersion curves of UiO-66 with correlated defects exhibit optical branches below 1 THz, indicating mode softening induced by the defects. We did not observe any imaginary frequencies in the defective structures, indicating that they are stable.

In the presence of missing linker correlated defects (bcu) in UiO-66, we observed changes in its acoustic phonons, which in turn affect its thermal conductivity. Specifically, we noted an increase in the slope of the longitudinal acoustic branch along the [010] direction compared to the pristine UiO-66 (Fig. 5b). This increase in slope indicates an increase in the group velocity in the *y*-direction, which could help to explain the unexpected rise in thermal conductivity observed in that direction. On the other hand, along the [100] and [001] directions, we observed the splitting of the transverse acoustic modes into two branches near the Brillouin zone center. One branch exhibited a lower slope, indicating a decrease in the group velocity (Fig. S10†). This finding is consistent with the observed decrease in thermal conductivity in the *x*- and *z*-directions compared to the pristine UiO-66. In the presence of missing cluster correlated defects (reo) in UiO-66, we observed a decrease in the slope of both the longitudinal and transverse acoustic branches compared to the pristine UiO-66 (Fig. 5c), and the dispersion relation was equivalent in all directions. This decrease in the slope of the acoustic branches is indicative of a decrease in the group velocity. Consequently, we observed a decrease in thermal conductivity by the same amount in all three directions in comparison to the pristine UiO-66. Finally, with scu-type missing cluster correlated defects, we observed transverse mode splitting in the [100] and [001] directions again (Fig. S12†). One branch had a very low slope, indicating a decrease in group velocity in the *x*- and *z*-directions compared to the reo structure. Conversely, the longitudinal acoustic branch had an increased slope along the [010] direction compared to the reo structure (Fig. 5d). This result may explain the observed increase in thermal conductivity in the *y*-direction and reduction in the *x*- and *z*-directions compared to the reo structure.

It should be noted that our assumption regarding the nanodomains within UiO-66 may not apply directly to bulk MOFs, such as the bcu analogues of UiO-66. While our assumption may not hold for bulk MOFs, we believe that our study provides valuable insights into the role of defects in shaping the thermal transport properties of MOFs in general, and our phonon band structure calculations provide a fundamental understanding of the lattice vibrations in UiO-66 and the effects of correlated defects on the phonon dispersion curves. The findings presented in this study may serve as a basis for further experimental studies and highlight the importance of defect engineering and its potential to manipulate the thermal transport properties of UiO-66 and other MOFs.

## Conclusion

In summary, we investigated the influence of missing linker and cluster defects on the thermal conductivity of a well-known MOF, UiO-66, using molecular dynamics simulation and the Green–Kubo method. Specifically, we first studied the dependency of thermal conductivity on the concentration of randomly incorporated defects. We found that both missing linker and cluster defects reduced the thermal conductivity of UiO-66, with the latter displaying a more pronounced effect in suppressing the heat flow. In addition to the density of defects, the spatial arrangement of defects was found to impact the thermal transport in UiO-66. This led us to explore the influence of various experimentally validated correlated defect nanoregions that followed three unique underlying topologies of bcu, reo, and scu nets. Surprisingly, the missing linker defects (33% defect density) that form correlated defect nanoregions (bcu) exhibited increased thermal conductivity (0.67 W m^−1^ K^−1^) in the *y*-direction compared to defect-free UiO-66 (0.32 W m^−1^ K^−1^), although the density decreased. The increase was driven by eliminating the phonon scattering source (perpendicular linkers) that inhibited thermal conductivity in the pristine UiO-66. Removing those linkers almost doubled the thermal conductivity in the perpendicular direction to the removed linkers but decreased it in the other two directions. In addition, we observed an increase in group velocity of acoustic phonons in the *y*-direction. This observation was also noticed in the correlated missing cluster defect nanodomain (scu topology). In this study, we demonstrated that controlled defects in MOFs can help impede thermal conductivity as well as enhance it by tuning their density and spatial order. Mainly for gas adsorption and catalysis applications, where defect sites provide better adsorption and active catalytic sites, using correlated missing linker defects (bcu topology) provides the additional privilege of better dissipating the exothermic heat out of the system.

## Data availability

The crystal structure data for all the pristine and defective UiO-66 structures used in this study are available in both CIF and LAMMPS file formats at https://github.com/meiirbek-islamov/defective-UiO-66. The code used to generate these defective structures, MOFUN, is described and referenced in the Methods section of the manuscript and is publicly available at https://github.com/WilmerLab/mofun. All other data relevant to this study are included in the manuscript or are available upon request from the corresponding author.

## Author contributions

M. I. performed the simulations, analyzed the results, and wrote the manuscript. P. B. helped to generate defective structures. H. B. and A. J. H. M. helped to perform phonon band structure calculations. C. E. W. supervised the research and contributed to the revision of the manuscript. All authors reviewed and edited the manuscript.

## Conflicts of interest

The authors declare the following competing financial interest(s): Christopher E. Wilmer has a financial interest in the start-up company NuMat Technologies, which is seeking to commercialize metal–organic frameworks.

## Supplementary Material

SC-014-D2SC06120A-s001
