# Supplementary material for: Correlated missing linker defects increase thermal conductivity in metal–organic framework UiO-66
Source: Chem Sci. 2023 May 31;14(24):6592–600. doi: 10.1039/d2sc06120a (PMC10284114; doi:10.1039/d2sc06120a)
Supplement: SC-014-D2SC06120A-s001 [file SC-014-D2SC06120A-s001.pdf]

Electronic Supplementary Material (ESI) for Chemical Science.
This journal is © The Royal Society of Chemistry 2023

Supporting Information

Correlated Missing Linker Defects Increase Thermal Conductivity in Metal-Organic Framework UiO-66

Meiirbek Islamov[1], Paul Boone[1], Hasan Babaei[2], Alan J. H. McGaughey[3], Christopher E. Wilmer*[1, 4]

[1]Department of Chemical & Petroleum Engineering, University of Pittsburgh, Pittsburgh, Pennsylvania 15261, United States

[2]Department of Chemistry, University of California, Berkeley, Berkeley, California 94720, United States

[3]Department of Mechanical Engineering, Carnegie Mellon University, Pittsburgh, Pennsylvania 15213, United States

[4]Department of Electrical & Computer Engineering, University of Pittsburgh, Pittsburgh, Pennsylvania 15261, Unites States

Included Sections

1. Thermal conductivity and correlation time plots for correlated defect nanoregions.

2. Relationship between thermal conductivity and defect concentration.

3. Relationship between mass density and defect concentration.

4. Phonon dispersion relations.

5. Vibrational density of states (DOS).

1. Thermal conductivity as a function of correlation time plots for correlated defect nanoregions.

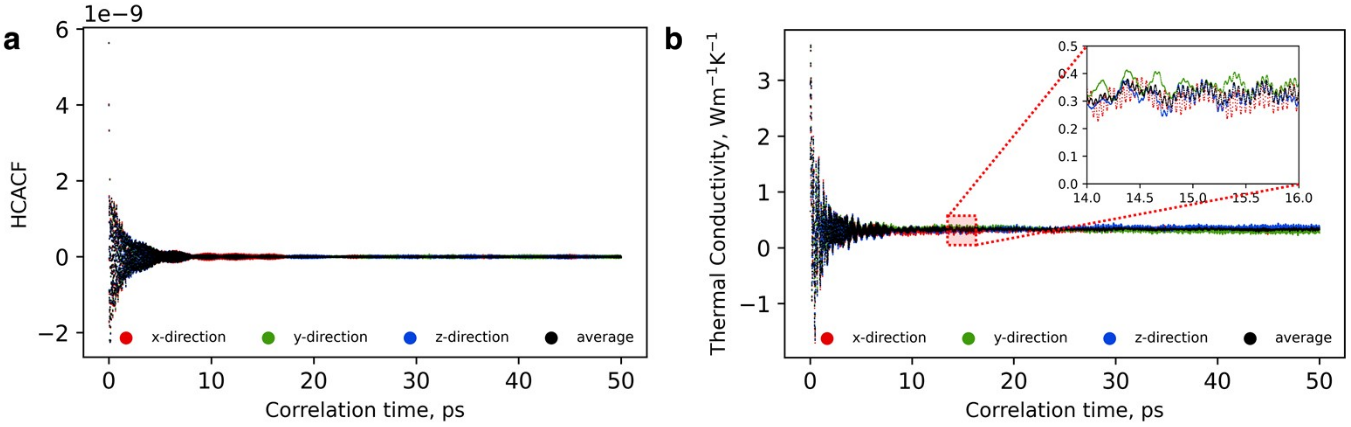

Figure S1. The HCACF **(a)** and thermal conductivity **(b)** plotted as a function of correlation time for pristine UiO-66 (3x3x3 supercells).

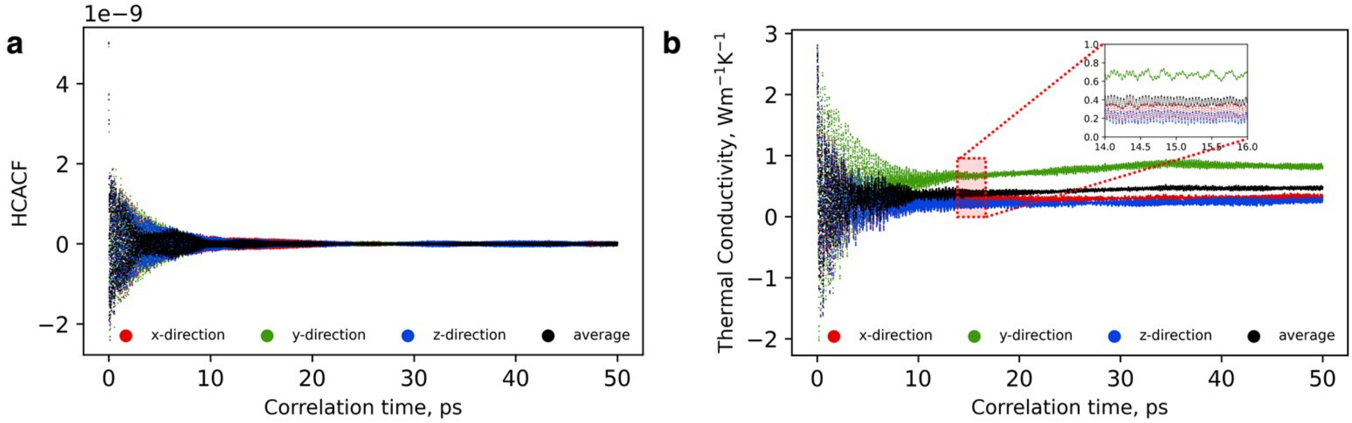

Figure S2. The HCACF **(a)** and thermal conductivity **(b)** plotted as a function of correlation time for missing linker correlated defect nanoregions (**bcu** topology).

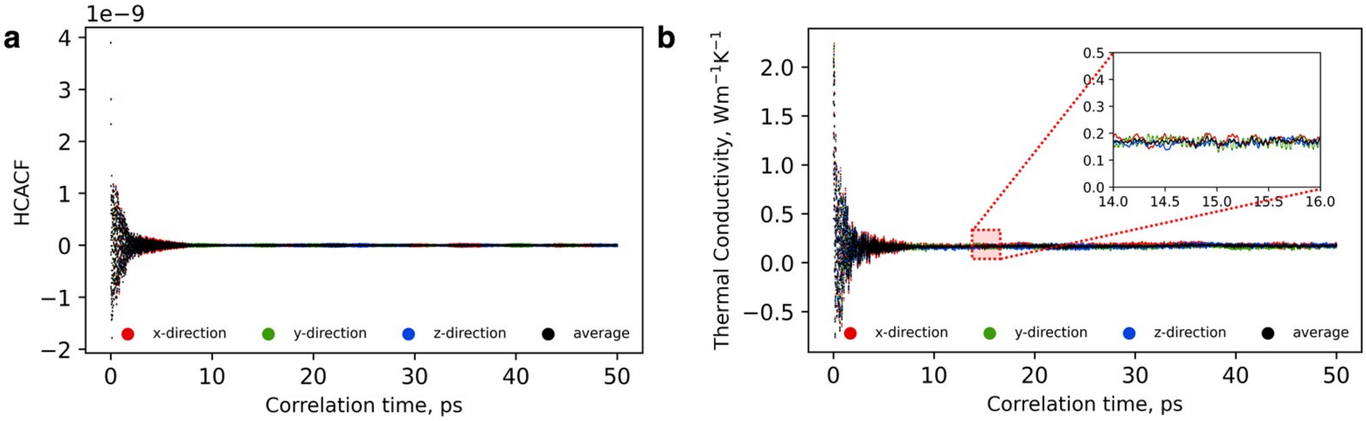

Figure S3. The HCACF **(a)** and thermal conductivity **(b)** plotted as a function of correlation time for missing cluster correlated defect nanoregions (**reo** topology).

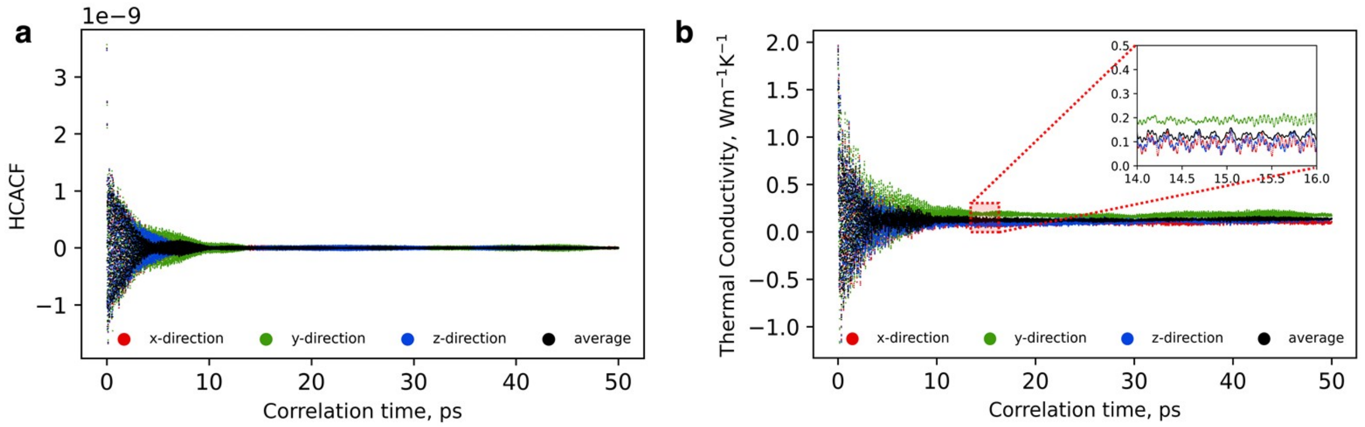

Figure S4. The HCACF **(a)** and thermal conductivity **(b)** plotted as a function of correlation time for missing cluster correlated defect nanoregions (**scu** topology).

2. Relationship between thermal conductivity and defect concentration

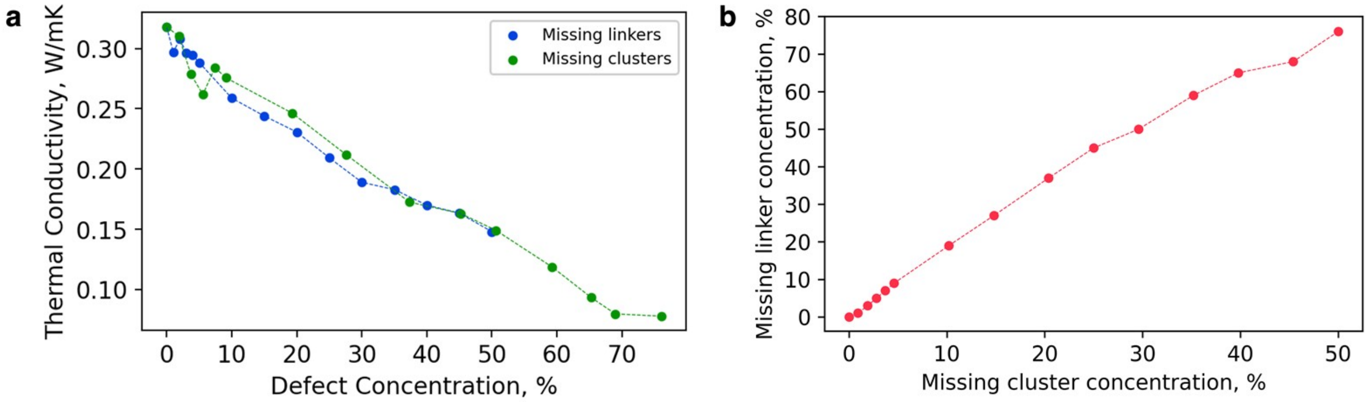

Figure S5. (a) Relationship between thermal conductivity and defect concentration, plotted against missing linker defect concentration as the x-axis, for UiO-66 with randomly incorporated missing linker and missing cluster defects (one replicate only). **(b)** Corresponding missing linker concentration as a function of missing cluster concentration for randomly incorporated missing cluster defects.

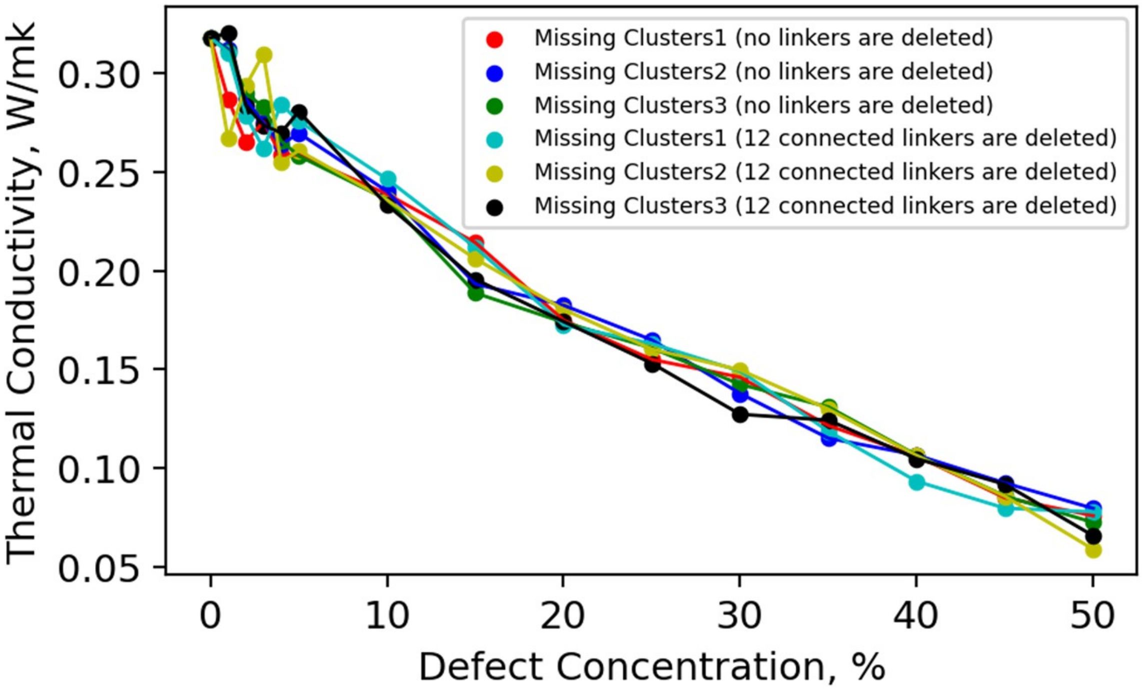

Figure S6. Relationship between thermal conductivity and randomly incorporated missing cluster defect concentrations, considering the deletion and non-deletion of all 12 linkers connected to the node.

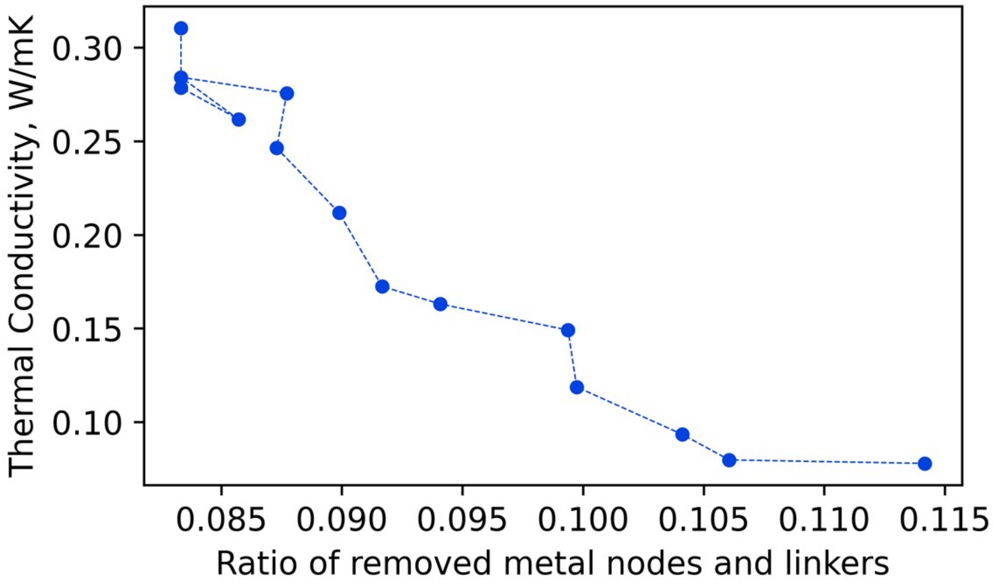

Figure S7. Relationship between thermal conductivity and the ratio of removed metal nodes to linkers for defective UiO-66 with randomly incorporated missing cluster defects.

3. Relationship between mass density and defect concentration.

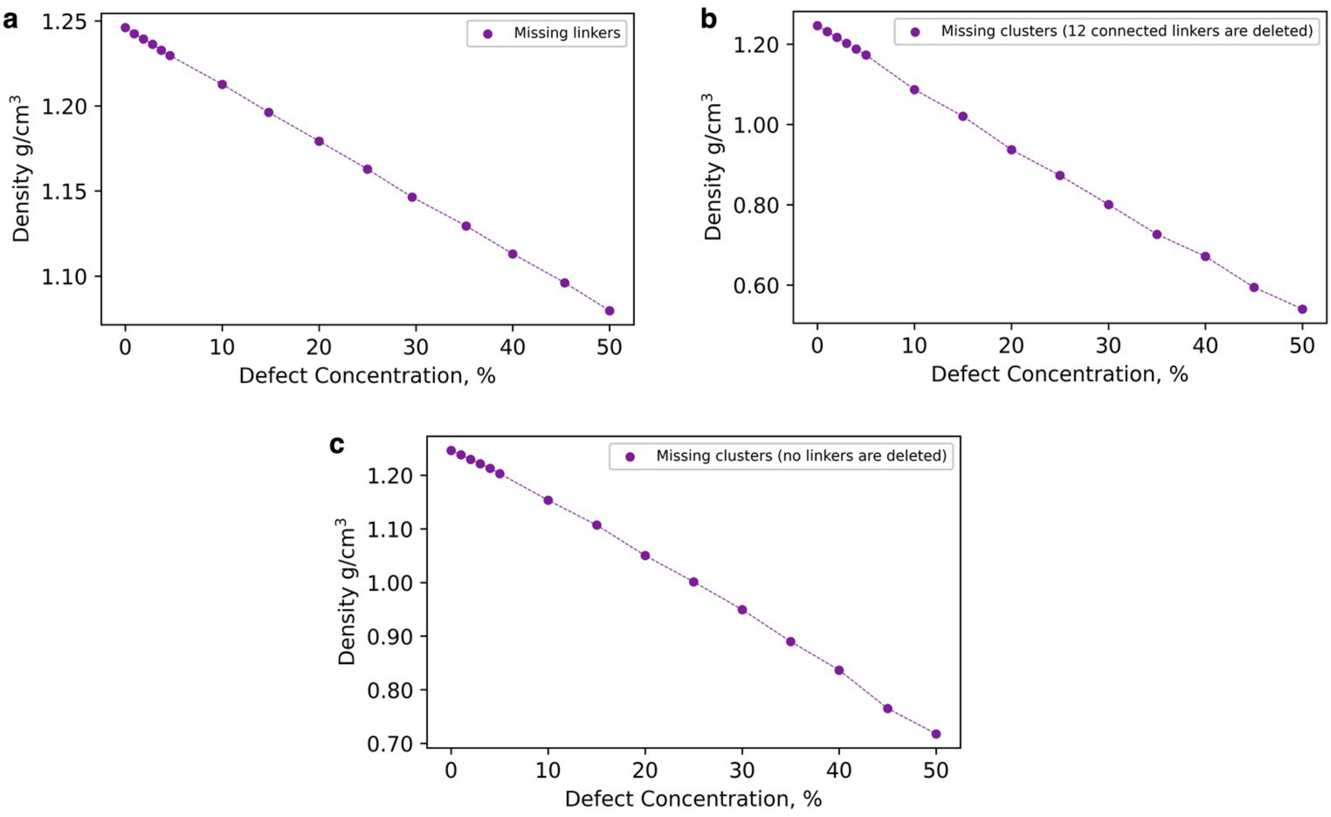

Figure S8. Relationship between mass density and defect concentration for UiO-66 with randomly distributed defects: **(a)** missing linker defects; **(b)** missing cluster defects where all connected 12 linkers are deleted; **(c)** missing cluster defects where no linkers are deleted.

4. Phonon dispersion relations.

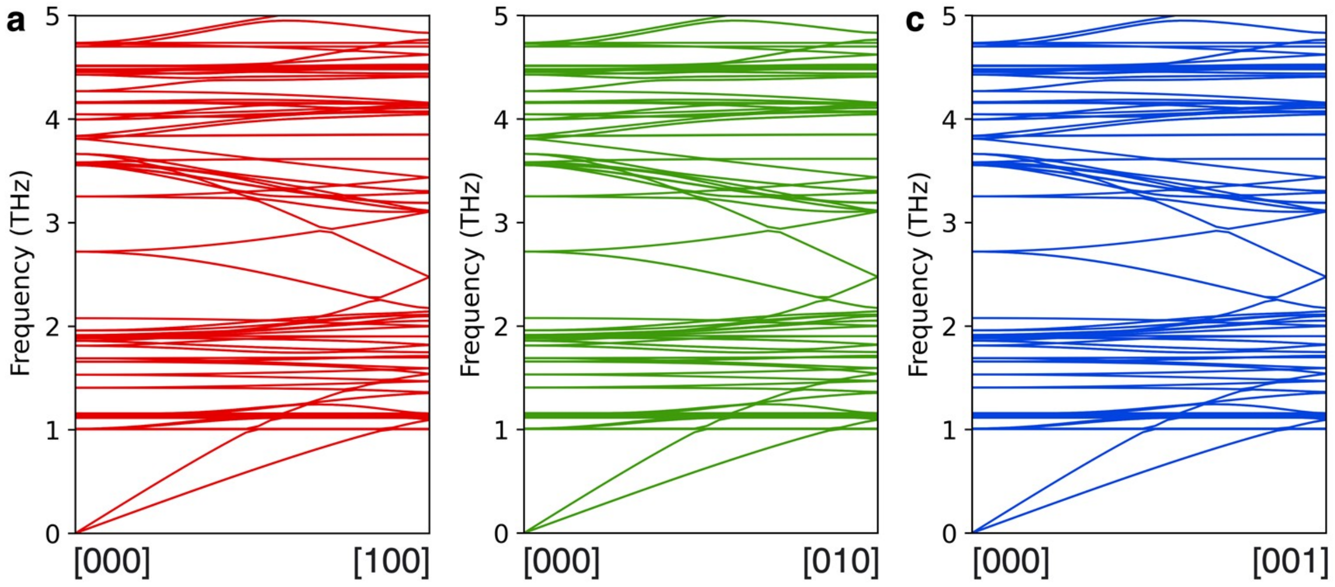

Figure S9. Phonon dispersion curves for pristine UiO-66.

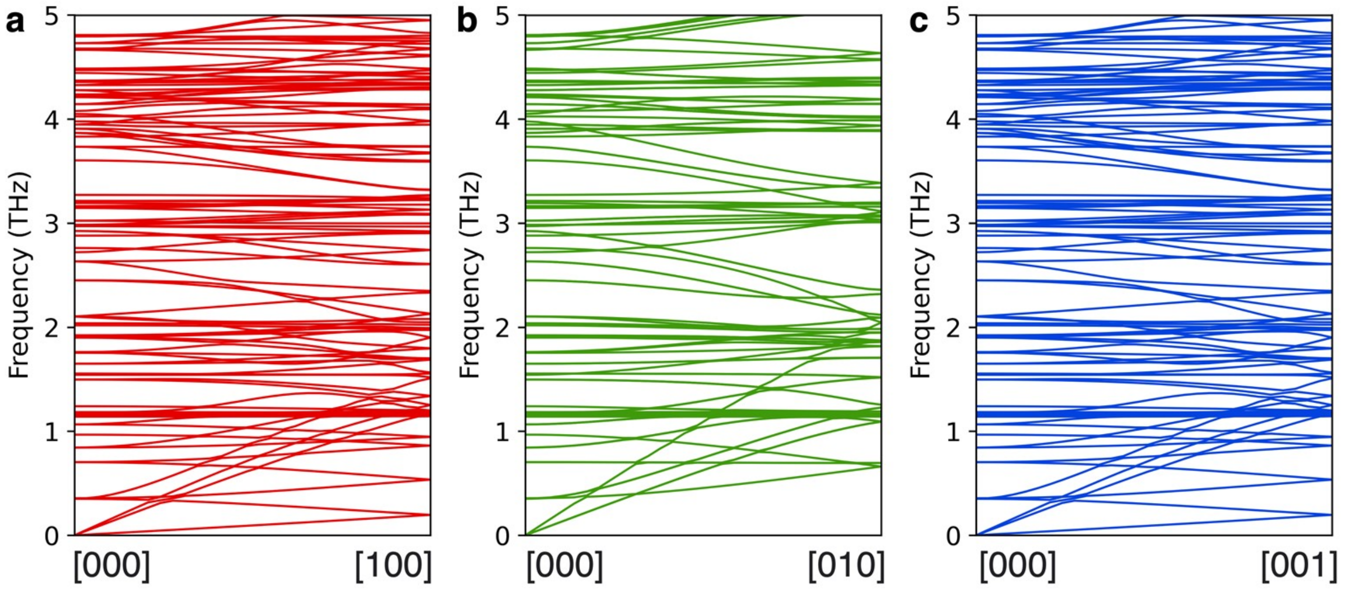

Figure S10. Phonon dispersion curves for defective UiO-66 with correlated missing linker defects (**bcu**).

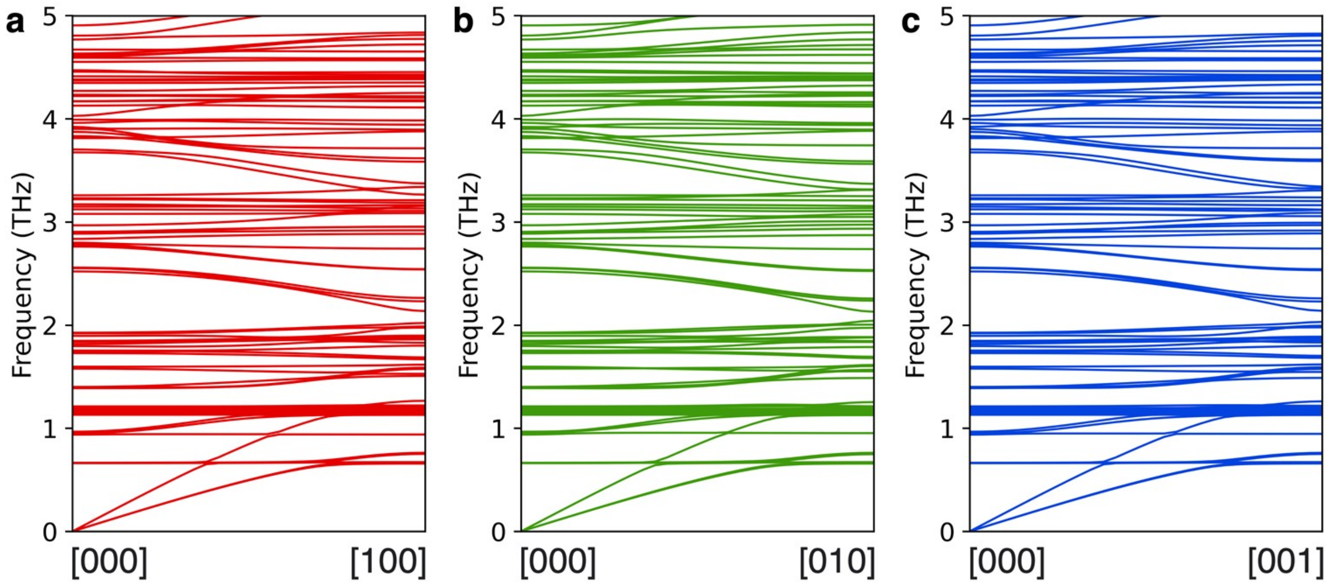

Figure S11. Phonon dispersion curves for defective UiO-66 with correlated missing cluster defects (**reo**).

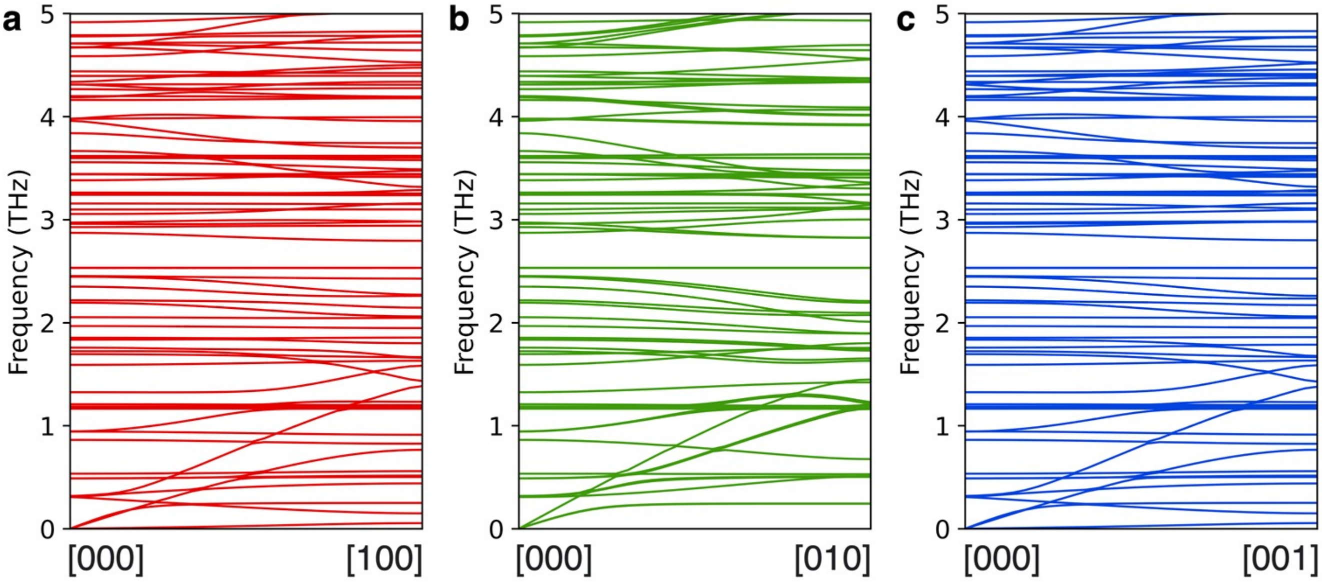

Figure S12. Phonon dispersion curves for defective UiO-66 with correlated missing cluster defects (**scu**).

5. Phonon density of states (DOS).

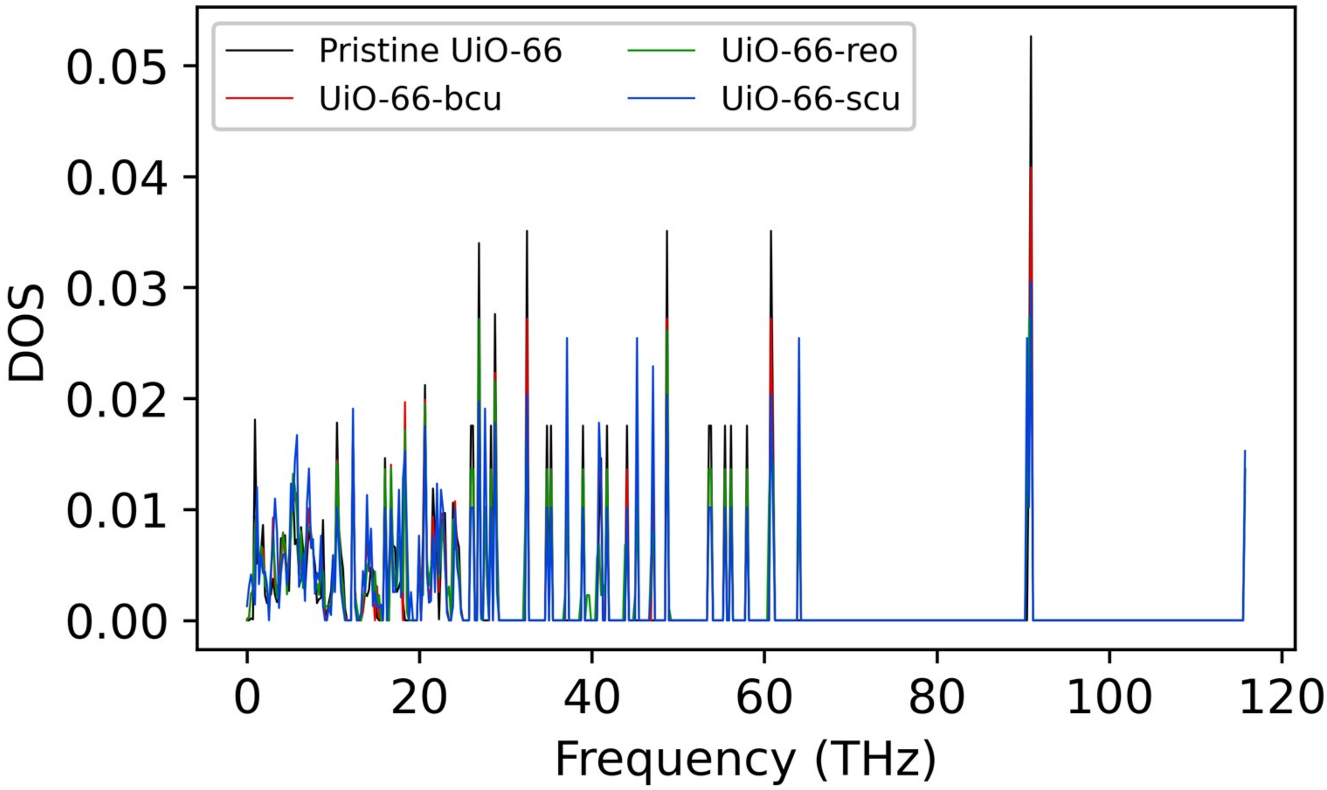

Figure S13. Vibrational density of states (DOS) for pristine UiO-66 and defective UiO-66 with correlated defects (**bcu**, **reo**, and **scu**).
